# Successful Topical Treatment of Hydroxyurea‐Induced Ulcers Without Drug Discontinuation: A Case Report

**DOI:** 10.1155/crh/5054123

**Published:** 2026-07-15

**Authors:** Yiming Wang, MinMin He, BoCheng Han, Qiwen Zhang, Rui Ma

**Affiliations:** ^1^ Department of Emergency, The People’s Hospital of Beilun District, Beilun Branch Hospital of The First Affiliated Hospital of Medical School Zhejiang University, 1288 Lushan East Road, Ningbo, Beilun District, 315800, China

**Keywords:** hydroxyurea, rb-bFGF, case report, skin ulcers

## Abstract

Hydroxyurea, an antineoplastic agent, is widely used to treat essential thrombocythemia and polycythemia vera. Although hydroxyurea‐induced skin ulcers are uncommon, they are a recognized long‐term adverse effect. Current management generally recommends discontinuation of hydroxyurea as the mainstay of treatment; however, drug withdrawal may aggravate the underlying hematologic disease. We report a case of bilateral medial malleolar ulcers associated with hydroxyurea therapy in a 65‐year‐old man with essential thrombocythemia, who showed marked improvement after two months of topical treatment with recombinant bovine basic fibroblast growth factor (rb‐bFGF) gel, without discontinuation of hydroxyurea. Complete healing was confirmed at 25‐month follow‐up, while hematologic disease control was maintained. This case suggests that localized wound care with topical rb‐bFGF may represent a feasible therapeutic option in selected patients when hydroxyurea discontinuation is not feasible.

## 1. Introduction

Hydroxyurea is a cytostatic agent that inhibits the S phase of the cell cycle, suppresses DNA synthesis, and generates cytotoxic free radicals [1, 2]. It is a widely used cytoreductive treatment option for essential thrombocythemia and polycythaemia vera. However, long‐term hydroxyurea therapy may lead to several adverse effects, such as mucocutaneous reactions, leg ulcers, and hydroxyurea‐related fever. Cutaneous ulcers are recognized but uncommon long‐term adverse effects and often improve within several months after discontinuation of the drug. Skin grafting has also been reported as an effective treatment for hydroxyurea‐induced ulcers, provided that hydroxyurea therapy is stopped. Nevertheless, some patients are unable to discontinue hydroxyurea because of disease severity or limited access to alternative agents such as interferon or ruxolitinib, owing to limitations in local healthcare resources or financial constraints. Here, we report a case of bilateral medial malleolar ulcers associated with hydroxyurea therapy that showed marked improvement after 2 months of topical treatment without discontinuation of hydroxyurea.

## 2. Case Report

### 2.1. Clinical History

A 65‐year‐old man with essential thrombocythemia presented to our outpatient clinic with refractory ulcers over both medial malleoli. He had been receiving hydroxyurea (1.0 g/day for 9 months, followed by 0.75 g/day for 5 months; cumulative dose: 368.75 g). After 14 months of therapy, he developed bilateral medial malleolar ulcers. Before presentation to our clinic, the patient had been treated with cephalosporins for 2 weeks at a local hospital for suspected skin infection; however, the ulcers did not improve. Hydroxyurea was continued after consultation with the hematologist because of persistently elevated platelet counts. His medical history included hypertension for 5 years. He had no history of diabetes mellitus, peripheral vascular disease, known allergies, psychiatric comorbidities, or related ulcers.

### 2.2. Examination/Diagnosis

**FIGURE 1:**
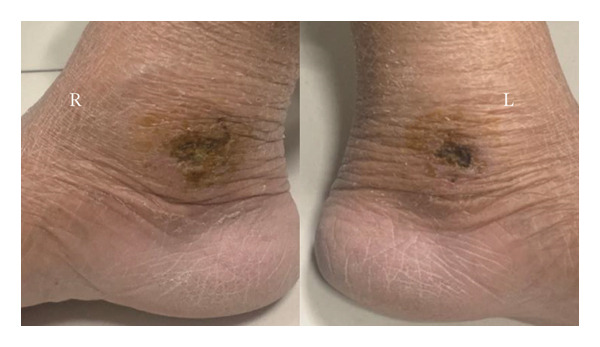
Two ulcers, each approximately 3 cm in diameter, were located on the medial aspect of both malleoli. The ulcer surfaces were covered with dry, black–brown eschar and showed no obvious exudate.

Two approximately 3‐cm ulcers were present, one on each medial malleolus. The lesions were covered with dry, blackish‐brown eschar and showed no exudation (Figure 1). Palpation revealed no fluctuance or granulation tissue but marked tenderness. Pain intensity was rated 3/10 at rest and 6/10 during dressing changes. At baseline, the white blood cell count was 7.5 × 10^9^/L, hemoglobin was 146 g/L, platelet count was 395 × 10^9^/L, and C‐reactive protein was 1.2 mg/L. Arterial and venous Doppler ultrasonography, as well as computed tomographic angiography of the lower extremities, showed no abnormalities. Differential diagnoses including infection, vasculitis, and malignancy were considered. Infection was considered unlikely because the patient had no clear clinical evidence of active infection, the C‐reactive protein level was normal, and the ulcers did not improve after 2 weeks of cephalosporin therapy. Vasculitis was considered less likely based on the clinical presentation and lack of supportive clinical findings. A skin biopsy was not performed; therefore, malignancy could not be completely excluded histopathologically. However, the overall clinical findings supported the diagnosis of hydroxyurea‐induced leg ulcers.

### 2.3. Treatment

The patient continued hydroxyurea therapy after discussion with the hematologist. The ulcers had been present for approximately 1 month before initiation of topical treatment. After cleansing the ulcer surface, recombinant bovine basic fibroblast growth factor (rb‐bFGF) gel was applied topically in a thin layer to completely cover the wound bed, at the recommended dose of 300 IU/cm^2^ according to the manufacturer’s instructions, followed by coverage with a foam dressing. Dressings were changed once daily for 2 months. No surgical or sharp debridement was performed, and no oral or intravenous antibiotics were administered during treatment. During treatment, serial complete blood counts remained stable, with white blood cell counts ranging from 7.0 to 7.6 × 10^9^/L and platelet counts ranging from 332 to 363 × 10^9^/L.

### 2.4. Outcomes

**FIGURE 2:**
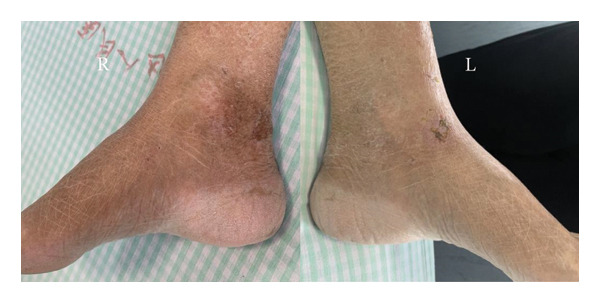
After 2 months of continuous dressing changes, both ulcers showed marked improvement with near‐complete healing. A new superficial ulcer developed adjacent to the left malleolus but remained stable without progression.

**FIGURE 3:**
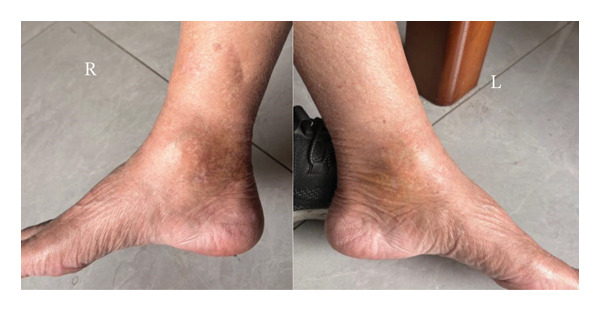
Long‐term follow‐up photographs obtained 25 months after treatment, showing complete healing of the bilateral medial malleolar ulcers, with residual postinflammatory hyperpigmentation and scarring but no obvious recurrence.

After 2 months of continuous dressing changes with daily topical rb‐bFGF application, both ulcers showed marked reduction in size with near‐complete healing (Figure 2). A new superficial ulcer developed adjacent to the left malleolus but showed no progression. At 25‐month follow‐up after treatment, the bilateral medial malleolar ulcers had completely healed, with full epithelialization and residual postinflammatory hyperpigmentation, without obvious recurrence on visual examination (Figure 3). At the most recent follow‐up, the patient remained on oral hydroxyurea at a dose of 1.0 g/day, and complete blood counts remained stable, with a white blood cell count of 8.0 × 10^9^/L and a platelet count of 321 × 10^9^/L.

## 3. Discussion

Hydroxyurea is the preferred cytoreductive agent for treating myeloproliferative neoplasms (MPNs), including polycythemia vera and essential thrombocythemia. According to the modified European LeukemiaNet (mELN) criteria, hydroxyurea intolerance is defined as nonhematological toxicities at any dose—including leg ulcers, mucocutaneous manifestations, gastrointestinal symptoms, pneumonitis, or fever—or failure to control disease‐related symptoms [3].

It has been reported that hydroxyurea intolerance occurs in 3.4% (5/148) of patients according to the ELN criteria and in 5.6% (8/148) of patients according to the mELN criteria [4]. Regarding skin toxicity, 8%–10% of patients develop refractory lower limb ulcers after approximately 1 year of therapy, typically during remission of essential thrombocythemia, a finding consistent across larger cohort studies [5, 6]. A prospective study by Stegelmann et al. in 172 MPN patients confirmed a significant association between hydroxyurea and the development of cutaneous adverse events, including ulcerous lesions, demonstrating that these complications are likely underestimated in retrospective series [7]. Since its first description in 1985, skin ulcers have become an increasingly recognized adverse effect [8].

Hydroxyurea‐induced ulcers appear to be more common in females than in males. Reported risk factors include venous insufficiency, arterial insufficiency, diabetes, and hypertension. It is important to note that the pathogenesis of leg ulcers in patients with MPNs receiving hydroxyurea is likely multifactorial: The underlying myeloproliferative disorder itself may contribute to ulcer formation through thrombocytosis‐related microvascular dysfunction, independent of hydroxyurea exposure [9]. Additionally, varicosity and other vascular comorbidities may independently cause or exacerbate ulceration. When ET‐related microvascular disease or venous insufficiency is identified as a contributing etiology, additional treatment options—including aspirin, compression therapy, and cytoreduction targeted at platelet normalization—become available [10]. Other causes of leg ulcers should be carefully excluded, as cases of chronic venous ulcers and vena saphena parva insufficiency mimicking hydroxyurea‐induced ulcers have been reported [11, 12].

Although the mean cumulative hydroxyurea dose associated with ulcer formation has been reported as 1733 g (range, 510–3960 g) [8] and 1533 g (range, 15–7520 g) [7], our patient developed ulcers after a cumulative dose of only 368.75 g. A possible explanation is that his 5‐year history of hypertension may have acted as an additional risk factor, accelerating the disease process.

Hydroxyurea‐induced ulceration is thought to be multifactorial. Hydroxyurea inhibits ribonucleotide reductase, leading to depletion of intracellular deoxyribonucleotide pools and suppression of DNA synthesis [1, 2]. As a result, cells in the S phase of the cell cycle are particularly susceptible, and rapidly proliferating cells such as basal keratinocytes may be damaged, thereby impairing keratinocyte turnover and epidermal regeneration [6, 13, 14]. The drug also acts as a free radical nitroxide in vivo, leading to chronic oxidative stress in the replicative basal keratinocyte layer and impairing nucleotide excision repair [15]. Long‐term exposure may also inhibit collagen formation and exert cytotoxic effects on vascular endothelial cells, impair angiogenesis, and disturb the microcirculation, ultimately contributing to cutaneous atrophy and delayed wound healing [16]. However, the exact pathogenesis remains incompletely understood. Ulcers commonly occur around the medial malleolus due to its anatomical vulnerability: Perforator veins in this region are prone to venous reflux, causing local stasis, tissue hypoxia, and chronic inflammation. The thin soft tissue overlying the bony prominence further impairs wound healing, and mechanical friction from footwear may exacerbate preexisting skin damage [17].

A characteristic feature of hydroxyurea‐induced ulcers is their painful and refractory nature—lesions show minimal response to conventional treatment and are often associated with chronic rest pain. Persistent nociceptor stimulation may promote the release of substance P [18, 19], thereby increasing local inflammation and vascular permeability, which perpetuates tissue injury [20, 21]. Topical opioids have been suggested not only to alleviate pain but also to promote wound healing. Chronic pain and recurrent ulcers can lead to psychological distress, including depression, social withdrawal, and even thoughts of amputation.

Previous case reports and case series have shown that hydroxyurea‐induced leg ulcers often improve only after withdrawal of hydroxyurea, typically within 3–6 months [6, 22, 23]. Wan et al. recently reported a case of recurrent hydroxyurea‐induced leg ulcers treated with a posterior tibial artery perforator propeller flap in a patient who continued hydroxyurea, with no recurrence in the flap area during 2‐year follow‐up, although a new ulcer developed contralaterally [24]. However, discontinuation may aggravate the underlying thrombocythemia and may not always be feasible in clinical practice. For patients meeting ELN criteria for hydroxyurea intolerance, recommended second‐line cytoreductive options include pegylated interferon‐α and busulfan; anagrelide represents an additional alternative, particularly for platelet control [25]. In contrast to the existing literature, hydroxyurea was continued in our patient because he declined switching to alternative therapy for financial reasons, and continued disease control was required. A topical regimen was initiated using rb‐bFGF gel, applied daily for 2 months. To our knowledge, there have been no previous reports describing the use of rb‐bFGF for hydroxyurea‐induced chronic skin ulcers. Rb‐bFGF is a well‐characterized member of the FGF family that binds heparan sulfate proteoglycans and activates FGF receptors (FGFRs), triggering downstream Ras/MAPK and PI3K/Akt signaling pathways. In this context, rb‐bFGF may help counteract hydroxyurea‐induced tissue damage through FGFR‐mediated repair signaling, promoting fibroblast proliferation and survival, extracellular matrix and collagen deposition, endothelial‐cell activation and angiogenesis, and granulation tissue formation [26, 27]. Topical rb‐bFGF has been shown in a large randomized controlled trial to significantly accelerate granulation tissue formation and epidermal regeneration, reducing healing time in burns and chronic dermal ulcers compared with placebo [28]. A 2024 retrospective study further demonstrated that rb‐bFGF gel significantly reduced wound healing time and pain scores in refractory wounds compared with conventional dressing changes alone [29]. In parallel, rb‐bFGF may facilitate keratinocyte migration and re‐epithelialization, thereby accelerating tissue reconstruction and wound closure [30]. Recent reviews of emerging wound‐healing therapies have highlighted the role of growth factors—including FGF‐2—in modulating the impaired microenvironment of chronic ulcers characterized by excessive protease activity and sustained inflammation [31, 32].

However, the existing literature on hydroxyurea‐induced ulcer management has largely relied on drug withdrawal as a prerequisite for healing. Hwang AS et al. reported two cases of recalcitrant hydroxyurea‐induced foot ulcers successfully treated with topical timolol [33]. However, both patients had discontinued hydroxyurea 2 months before treatment, making it difficult to determine whether healing was attributable to drug withdrawal or timolol. Similarly, Akinci et al. described a case in which hyperbaric oxygen therapy improved hydroxyurea‐associated leg ulcers [34]; however, that patient had also discontinued hydroxyurea. A 2024 case report from India documented ulcer healing in a sickle cell disease patient only after hydroxyurea discontinuation [35]. Across these cases, the contribution of the adjunctive intervention remains difficult to isolate from the effect of drug withdrawal itself.

The present case differs critically from this prior experience. Hydroxyurea was neither discontinued nor replaced with an alternative cytoreductive agent throughout the entire treatment course; instead, marked and sustained ulcer healing was achieved with adjunctive local wound care and topical rb‐bFGF while hydroxyurea was continued. Complete wound closure was maintained at 25‐month follow‐up, representing the first reported case, to our knowledge, of hydroxyurea‐induced ulcer healing without drug discontinuation.

This observation carries potential clinical significance, particularly for patients in whom cytoreductive therapy cannot safely be withheld—such as those with high‐risk essential thrombocythemia or polycythemia vera requiring ongoing disease control. Topical rb‐bFGF may represent a viable adjunctive strategy in such scenarios. Nevertheless, this finding must be interpreted cautiously, as it is derived from a single case, and prospective studies are needed to confirm the efficacy and safety of this approach.

## 4. Conclusion

Skin ulcers represent a recognized and potentially refractory adverse effect of long‐term hydroxyurea therapy, with a cumulative dose‐dependent relationship that may be modified by individual comorbidities. This case suggests that in selected patients for whom discontinuation of hydroxyurea or switching to alternative cytoreductive therapy is not feasible, topical rb‐bFGF combined with local wound care may represent a practical therapeutic option. In our patient, marked ulcer improvement was observed after 2 months of treatment, and complete healing was confirmed at 25‐month follow‐up despite continued hydroxyurea therapy, without obvious recurrence and with maintained hematologic disease control. Further prospective studies and larger case series are needed to define the role of localized therapy in the management of hydroxyurea‐associated ulcers.

## 5. Limitations and Future Directions

This report has several limitations. It describes a single patient without a control comparator, and spontaneous improvement cannot be entirely excluded, although this appears less consistent with published evidence in which complete healing without hydroxyurea withdrawal is uncommon. Serial wound‐area measurements and histopathologic confirmation were not obtained. Future studies should include larger case series and prospective or comparative trials. Long‐term surveillance data on ulcer recurrence in patients who continue hydroxyurea without dose reduction are particularly needed.

## Author Contributions

Yiming Wang (first author): project development, and manuscript writing and editing. MinMin He: project development. BoCheng Han and Qiwen Zhang: manuscript editing. Rui Ma (corresponding author): project development.

## Funding

This research did not receive any specific grant from funding agencies in the public, commercial, or not‐for‐profit sectors.

## Disclosure

All authors have read and approved the final version of the manuscript. The authors confirm that no persons or third‐party services who are not listed as authors were involved in the research. Dr. Rui Ma had full access to all of the data in this study and takes complete responsibility for the integrity of the data and the accuracy of the data analysis.

## Ethics Statement

The Institutional Ethics Committee of Beilun District People’s Hospital reviewed the study protocol and confirmed that it met the criteria for exemption from ethical review in accordance with the Declaration of Helsinki.

## Consent

Written informed consent was obtained from the patient for publication of this case report and the accompanying clinical photographs.

## Conflicts of Interest

The authors declare no conflicts of interest.
